# Death of Joshua Brookes

**Published:** 1833-02

**Authors:** 


### OBITUARY.

### DEATH OF JOSHUA BROOKES, ESQ., F.R.S., F.L.S., SOC. CÆS. NAT. CUR. MOSQ. SOC.

This gentleman died suddenly, on the 10th January, at his residence in Great Portland street. For nearly fifty years Mr. Brookes was professor of anatomy to a very numerous class of pupils, whose esteem he always merited, for the patient and even laborious assiduity with which he endeavoured to keep them to their studies, and to perfect them in their knowledge of anatomy. Few, if any, anatomical teachers in this country have ever given up so much time, or evinced such unwearied zeal, as Mr. Brookes always did, during his very long career as a teacher to the fatiguing and by no means agreeable business of the dissecting room; for there he might almost constantly be seen, watching over and guiding the progress of the young anatomists around him. Mr. Brookes' style of delivering his lectures did not, perhaps, rise to eloquence, but he was always fluent, clear, and unaffected, in his mode of delivery: his meaning could never be mistaken by the dullest of his auditors. He was, in fact, an excellent teacher of anatomy, in every sense of the word. We profited by his instructions for several years, and we speak, therefore, from personal experience, as well as from the common opinion of those who were then commencing their studies with us.

It is well known that Mr. Brookes formed, by unexampled industry and perseverance, the most splendid and extensive collection of anatomical and zoological specimens ever accumulated in this or any other country, with the exception, perhaps, of that of John Hunter. A few years ago, a great part of Mr. Brookes' museum was sold by public auction, at a considerable loss. Many of the most valuable specimens, it is true, were purchased for foreign collections at high prices, but the average return was lamentably deficient. We know not whether such a hint was ever thrown out at the time, but we think, if an attempt had been made to purchase the museum by a very moderate subscription, that the public, as well as the profession, would have supported the undertaking, and that we might now have had a durable monument to the memory of one of our best practical anatomists and scientific zoologists.

